# Deubiquitinase USP35 modulates ferroptosis in lung cancer via targeting ferroportin

**DOI:** 10.1002/ctm2.390

**Published:** 2021-05-01

**Authors:** Zheng Tang, Wanli Jiang, Ming Mao, Jinping Zhao, Jiakuan Chen, Nitao Cheng

**Affiliations:** ^1^ Department of Thoracic Surgery Zhongnan Hospital of Wuhan University Wuhan China; ^2^ Department of Thoracic Surgery Renmin Hospital of Wuhan University Wuhan China

**Keywords:** ferroportin, ferroptosis, lung cancer, ubiquitin‐specific protease 35

## Abstract

**Background:**

Ferroptosis is essential to regulate tumor growth and serves as a promising therapeutic target to lung cancer. Ubiquitin‐specific protease 35 (USP35) belongs to the deubiquitinases family that is associated with cell proliferation and mitosis. In this research, we aim to elucidate the potential role and molecular basis of USP35 in lung cancer.

**Methods:**

Lung cancer cells were infected with lentiviral vectors to silence or overexpress USP35. Cell viability, colony formation, lipid reactive oxygen species production, intracellular iron metabolism, and other ferroptotic markers were detected. The role of USP35 on ferroptosis and tumor progression were also tested in mouse tumor xenograft models in vivo.

**Results:**

USP35 was abundant in human lung cancer tissues and cell lines. USP35 knockdown promoted ferroptosis, and inhibited cell growth, colony formation, and tumor progression in lung cancer cells. USP35 overexpression did not affect tumorigenesis and ferroptosis under basal conditions, but reduced erastin/RSL3‐triggered iron disturbance and ferroptosis, thereby facilitating lung cancer cell growth and tumor progression. Further studies determined that USP35 directly interacted with ferroportin (FPN) and functioned as a deubiquitinase to maintain its protein stability. More importantly, we observed that USP35 knockdown sensitized lung cancer cells to cisplatin and paclitaxel chemotherapy.

**Conclusion:**

USP35 modulates ferroptosis in lung cancer via targeting FPN, and it is a promising therapeutic target to lung cancer.

## Graphical abstract

1. USP35 is abundant in human lung cancer tissues and cell lines.

2. USP35 modulates iron homeostasis and ferroptosis in lung cancer tissues and cell lines.

3. USP35 directly interacts with ferroportin and maintain its protein stability to prevent iron overload and ferroptosis.

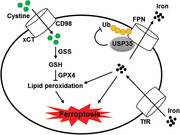

## 1 INTRODUCTION

Lung cancer remains the leading cause of cancer‐associated deaths all over the world that evokes great burden to the economic and healthcare system. Despite the advances of chemotherapeutic drugs and novel antitumor strategies, the long‐term outcome of these patients remains poor.^1^, ^2^, ^3^ Thus, it is urgently needed to dissect the pathogenesis of lung cancer and subsequently find effective therapeutic targets.

Ferroptosis is a novel type of regulated cell death that morphologically, biochemically and genetically differs from classical necrosis, apoptosis, or autophagy.^4^, ^5^ Emerging researches demonstrate that ferroptosis is essential to regulate tumor growth, including lung cancer, and that targeting ferroptosis may provide novel insights in lung cancer treatments.^6^, ^7^, ^8^ It is well‐accepted that ferroptotic cell death involves iron‐dependent accumulation of toxic lipid‐based reactive oxygen species (ROS) tightly linked with glutathione (GSH) and iron metabolism.^9^ Cystine serves as a rate‐limiting substrate for GSH synthesis and its acquisition requires cystine‐glutamate antiporter (system *x*_c_^−^) to export intracellular glutamate, which then triggers GSH synthesis with glutamate and glycine under the control of glutathione synthetase (GSS).^10^ Glutathione peroxidase 4 (GPX4) functions as a GSH‐dependent antioxidant enzyme to catalyzes the reduction of reactive lipid hydroperoxides to non‐reactive lipid alcohols upon oxidative stress, thereby preventing polyunsaturated fatty acids (PUFAs) fragmentation and cell membrane damage.^11^, ^12^ In addition to the GSH/GPX4‐dependent ROS scavenging mechanism, iron metabolism and its associated ROS formation also contribute to the occurrence of ferroptosis. Intracellular iron homeostasis relies on the uptake of extracellular iron by transferrin (Tf)/transferrin receptor (TfR) system and ferroportin (FPN)‐mediated iron export.^13^, ^14^ Of note, FPN is the only discovered iron transport protein in mammals, and inhibiting FPN‐dependent iron export increases intracellular iron load and ferroptosis sensitivity.^15^ Collectively, these findings define the intracellular iron homeostatic network and ferroptosis as promising therapeutic targets to lung cancer.

Ubiquitination is a critical protein posttranslational modification and has versatile biological functions via controlling protein degradation. As expected, it is a reversible process similar to other post‐translational modifications that can be catalyzed by deubiquitinases (DUBs) via removing ubiquitin moieties from the ubiquitinated substrates.^16^, ^17^, ^18^ Ubiquitin‐specific proteases (USPs) belong to the DUBs family, and play indispensable roles in the malignant cell phenotypes and cancer progression due to their oncogenic or tumoricidal actions.^19^, ^20^ Moreover, multiple USPs have been determined as the promising therapeutic targets to lung cancer. Wang et al found that forced USP10 expression suppressed lung tumorigenesis, while loss of USP10 accelerated lung adenocarcinoma initiation and progression.^21^ Besides, USP22 promoted proliferation, migration, and invasion in lung adenocarcinoma cells, and contributed to the chemoresistance.^22^ Results from Yu et al suggested that USP14 inhibited gefitinib (GFB) sensitivity and that high USP14 level correlated with a perishing prognosis.^23^ USP35 is a member of the USPs family that has been associated with cell proliferation and mitosis.^24^, ^25^ A very recent study detected an upregulation of USP35 in ovarian cancer tissues and proved that USP35 knockdown sensitized ovarian cancer cells to cisplatin (DDP).^26^ Yet, the potential role and molecular basis of USP35 in lung cancer remain unclear.

## 2 MATERIALS AND METHODS

### 2.1 Reagents

Necrostain‐1 (Nec‐1, #N9037; a necroptosis inhibitor), Z‐VAD‐FMK (Z‐VAD, #V116; a pancaspase inhibitor), chloroquine (CQ, #C6628; an autophagy inhibitor), GSH assay kits (#CS0206), MG132 (#M7449, a proteasome inhibitor), DDP (#P4394), paclitaxel (PTX, #1491332), and GFB (#SML1657) were purchased from Sigma‐Aldrich (St. Louis, MO, USA). Mdivi‐1 (#S7162), cyclosporine A (CsA, #S2286), liproxstatin‐1 (Lip‐1, #S7699), ferrostain‐1 (Fer‐1, #S7243), erastin (#S7242), and RSL3 (#S8155) were obtained from Selleck Chemicals (Houston, TX, USA). Mem‐PER™ Plus Membrane Protein Extraction Kit (#89842), NE‐PER™ nuclear and cytoplasmic extraction reagents (#78833), and BODIPY™ 581/591 C11 (BODIPY‐C11, #D3681) were purchased from Invitrogen (Carlsbad, CA, USA). Malondialdehyde (MDA) assay kits (#ab118970) and plasma membrane protein extraction kit (#ab65400) were from Abcam (Cambridge, UK). Cell counting kit‐8 (CCK‐8, #CK04) was purchased from Dojindo Laboratories (Tokyo, Japan). Lentiviral vectors carrying the short hairpin RNA against human USP35 (sh*USP35*) or the scramble control (sh*RNA*) were generated by Gene Pharma Corporation (Shanghai, China), and the sequence of sh*USP35* was provided as below: 5′‐GGGAAGATCTGATGATGTT‐3′. For USP35 overexpression, human USP35 cDNA (*USP35*) or a negative control (*CTRL*) sequence was cloned into the lentiviral vectors by Gene Pharma Corporation (Shanghai, China). Anti‐p21(#ab109520), anti‐p27 (#ab32034), anti‐cyclin D1 (#ab16663), anti‐xCT (#ab37185), anti‐CD98 (#ab108300), anti‐glutathione synthetase (GSS, #ab124811), anti‐GPX4 (#ab125066), anti‐Tf (#ab109503), anti‐TfR (#ab84036), anti‐sodium potassium ATPase (Na^+^‐K^+^ ATPase, #ab76020), anti‐β‐actin (#8226), anti‐USP35 (#ab86791), and anti‐ubiquitin (Ub, #ab134953) were from Abcam (Cambridge, UK), while anti‐FPN (#NBP1‐21502) was obtained from Novus Biologicals (Littleton, Colorado, USA). Anti‐p‐phosphatidylinositol 3 kinase (p‐PI3K, #4228), anti‐t‐PI3K (#4257), anti‐p‐protein kinase B (p‐AKT, #4060), anti‐t‐AKT (#4691), anti‐p‐MAP kinase kinase 1/2 (p‐MEK1/2, #9154), anti‐t‐MEK1/2 (#9122), anti‐p‐extracellular signal‐regulated kinase 1/2 (p‐ERK1/2, #4370), and anti‐t‐ERK1/2 (#4695) were purchased from Cell Signaling Technology (Beverly, MA, USA).

### 2.2 Cell lines and treatments

The normal human lung epithelial cell lines (BEAS‐2B and HBE) and lung cancer cell lines (A549, H358, H460, H1299 and H1650) were obtained from ATCC, and maintained in RPMI 1640 medium containing 10% FBS.^27^, ^28^ The cells were incubated with sh*USP35* for 12 h at a multiplicity of infection (MOI) of 20 to knock down endogenous USP35 expression or sh*RNA* as a negative control. For USP35 overexpression, the cells were incubated with *USP35* at a MOI of 10 or the *CTRL* for 12 h.^29^ The cells were subsequently cultured in normal medium containing 10% FBS for additional 96 hours except special annotation. To inhibit necrosis, apoptosis, autophagy, mitophagy, and ferroptosis, the cells were treated with Nec‐1 (10 µmol/L), Z‐VAD (10 µmol/L), CQ (25 µmol/L), Mdivi‐1 (15 µmol/L), CsA (5 µmol/L), Fer‐1 (1 µmol/L) or Lip‐1 (0.2 µmol/L) for 96 h after the removal of lentiviral vectors.^29^, ^30^, ^31^ To neutralize the excessive iron or ROS, membrane permeable (CPX, 5 µmol/L) and impermeable (DFO, 100 µmol/L) iron chelators and GSH (1 mmol/L) were used.^30^, ^32^, ^33^ Besides, the cells were incubated with erastin (5 µmol/L) or RSL3 (2 µmol/L) for 96 h after the removal of lentiviral vectors.^7^ To knock down the endogenous FPN expression, the cells were preinfected with sh*FPN* at a MOI of 10 or sh*RNA* for 12 h and then cultured in normal medium for 12 h before USP35 manipulation, erastin or RLS3 treatment. For proteasome inhibition, MG132 (20 µmol/L) was added at the last 12 h before experimental end.^34^ To investigate the role of USP35 in DDP, PTX, or GFB‐mediated chemosensitivity, sh*RNA* or sh*USP35*‐infected cells were incubated with DDP (20 µmol/L), PTX (0.3 µmol/L), or GFB (30 µmol/L) at the last 12 h before experimental end.^35^, ^36^, ^37^

### 2.3 Cell viability and colony formation measurements

CCK‐8 kits were used to measure cell viability and the absorbance at 450 nm was detected as the number of living cells.^38^, ^39^, ^40^ To detect cell colony formation, the cells were seeded onto the six‐well plates for 2 weeks, which were then stained with 0.1% crystal violet.^7^, ^41^ The cells were rinsed with tap water and dried at room temperature. Finally, the visible colonies were counted using the Image J software in a blinded manner with the colonies >0.05 mm included.

### 2.4 Cell cycle analysis

Cell cycle was analyzed by propidium iodide (PI) as previously described.^42^, ^43^, ^44^ In brief, the cells were fixed in 70% ethanol at ‐20°C overnight and then incubated with PI/RNase Staining Buffer (BD Bioscience) at 37°C for 30 min. Eventually, the DNA content was evaluated using the flow cytometry and quantified by Modfit software.

### 2.5 Transwell assay

Transwell assay was performed to determine the migrative and invasive capacities of lung cancer cells using Transwell cell inserts precoated with or without polymerized Matrigel as previously described.^45^, ^46^, ^47^ For migration analysis, cells at a density of 2 × 10^5^ were plated into the upper chamber without polymerized Matrigel, while 0.8 mL medium containing 20% FBS was added to the lower chamber. Then, the cells remaining in the upper chamber surface were gently wiped out using a cotton swab, while the cells in the bottom of the filters were stained with 0.1% crystal violet for 40 min at room temperature. For invasion analysis, the upper Transwell cell inserts were pre‐coated with polymerized Matrigel. The migrative and invasive cells were captured using a light microscope and randomly calculated under at least 5 fields in a blinded manner.

### 2.6 Lipid peroxidation measurements

Lipid peroxidation was evaluated by the lipid ROS level and MDA content according to previous studies.^7^, ^48^ Briefly, the cells were stained with C11‐BODIPY (10 µmol/L, 37°C for 30 min) in the dark, and then were exposed to the flow cytometry analysis at 484/510 nm and 581/610 nm to examine the amount of intracellular ROS level. MDA concentration in cell lysates was detected by a commercial kit at 532 nm according to the manufacturers’ instructions.

### 2.7 Hydroxyeicosatetraenoic acid detections

The levels of hydroxyeicosatetraenoic acids (HETEs) released to the medium were detected by LC‐MS/MS to assess cell membrane damage by lipid peroxidation according to previous studies.^11^, ^49^ Briefly, cell medium was collected and centrifuged to obtain the cell‐free supernatants, which were then mixed with PGE2‐d4 and 15‐HETE‐d8 for internal adjustment. Next, the lipid was extracted to the hexane layer and dried under vacuum. Finally, the amounts of 5‐, 11‐, 12‐, 15‐HETE were detected using LC‐MS/MS.

### 2.8 GSH level and GPX4 activity measurements

GSH level was quantified using the commercial kit, while the relative GPX4 activity was measured using a previously‐described substrate phosphatidylcholine hydroperoxide as a substrate.^12^, ^50^

### 2.9 Iron assay

Labile iron pool (LIP) was detected using calcein‐acetoxymethyl ester and DFO as previously described.^32^ Briefly, the cells were incubated with calcein (2 µmol/L, 37°C for 30 min) and then DFO (100 µmol/L) was added to remove iron from calcein. The fluorescence parameters was measured with excitation/emission at 485 nm/535 nm using a fluorescence BioTek plate reader, and the change of fluorescence intensity with or without DFO treatment indirectly quantified as the amount of LIP. Intracellular ferrous iron (Fe^2+^) levels were quantified with a commercial kit and the absorbance at 593 nm was calculated as the intracellular Fe^2+^ levels.

### 2.10 Immunoblots (IB) and immunoprecipitation (IP)

Proteins were extracted using RIPA lysis buffer and quantified by bicinchoninic acid method. Then, the proteins were separated by SDS‐PAGE and transferred onto the PVDF membranes, followed by the incubation with indicating primary antibodies and peroxidase‐conjugated secondary antibodies, respectively. The protein bands were visualized by chemiluminescence and quantified by the Image J software in a blinded manner.^51^, ^52^, ^53^ Membrane and nuclear protein were extracted using the commercial kits, and Na^+^‐K^+^ ATPase and Lamin B1 were regarded as the internal control.

For IP assay to detect the endogenous protein interactions, the primary antibodies and protein A/G agarose beads were added to the protein lysates and incubated at 4°C overnight. Then, the beads were boiled after extensive washing and separated on the SDS‐PAGE electrophoresis.^26^

### 2.11 Quantitative real‐time PCR

Total RNA was extracted using the RNeasy Mini kit according to the manufacturer's instructions and quantitative real‐time PCR was performed to detect relative gene expression using QuantiNova SYBR Green PCR Kit (Qiagen; Hamburg, Germany).

### 2.12 Cystine uptake assay

Cells were pre‐washed by Na^+^‐free uptake buffer for two times and then incubated in uptake buffer at 37°C for 10 min. Next, the cells were incubated in the uptake buffer containing ^14^C cystine (0.2 µCi/mL) for additional 10 min, which were then rinsed by the cold uptake buffer and lysed in NaOH (0.1 mol/L).^54^ The TRI‐CARB 4810TR 110 V Liquid Scintillation Counter (PerkinElmer; Shelton, CT, USA) was used to detect the radioactivity (disintegrations per minute, DPM).

### 2.13 Tumor xenografts

BALB/c nude mice (4‐5 weeks old) were obtained from HFK Bioscience Co., Ltd (Beijing, China) and maintained in a SPF barrier system. All animal experiments were performed in accordance with the ARRIVE guidelines and also approved by the Animal Ethics Committee of Zhongnan Hospital of Wuhan University. H460, H1299 or H1650 cell lines at a dose of 1 × 10^6^ with or without USP35 manipulation were subcutaneously injected into the right dorsal flank of the nude mice.^55^, ^56^, ^57^ These mice were monitored daily and the tumor volume was calculated every 5 days based on calliper measurements by the following formula: tumor volume = 1/2 length × width.^2^ To knock down FPN, the lung cancer cells were preinfected with sh*FPN* or sh*RNA* before transplanting to the BALB/c mice. To induce ferroptosis in vivo, the tumor‐bearing mice were treated with erastin (15 mg/kg twice every other day) by intraperitoneal injections or RSL3 (100 mg/kg twice a week) via intratumoral injections from day 18 after cell inoculation.^12^, ^58^ To validate the role of USP35 on the chemosensitivity of lung cancer cells, the tumor‐bearing mice were intraperitoneally treated with DDP (5 mg/kg) or PTX (15 mg/kg) three times a week from day 18 after cell inoculation.^59^ Besides, they were also orally treated with GFB (25 mg/kg/d) for the last 12 consecutive days to determine the synergistic effects between USP35 knockdown and EGFR inhibition.^60^ All mice were sacrificed at day 25 after cell inoculation with the tumor tissues collected for further study.

### 2.14 Human tumor samples

Human lung adenocarcinoma (ADC) tissues and squamous cell cancer (SCC) tissues were obtained from the patients without neoadjuvant or adjuvant therapies before surgery. Corresponding adjacent normal tissues (ANT) was obtained from the same patients at least 3 cm away from the tumor border. Written informed consent was obtained from the patients and families of the donors. All the experimental procedures involving human samples completely adhered to the ethical standards outlined in the Declaration of Helsinki and also approved by the Zhongnan Hospital of Wuhan University Review Board in Wuhan.

### 2.15 Statistical analysis

Data were represented as mean ± SD and analyzed by SPSS software. Unpaired Student's two‐tailed *t*‐tests were applied to compare the different means from two groups, whereas the comparisons among three or more groups were performed by the one‐way ANOVA followed by the Tukey post‐hoc test. Statistical significance was defined as *P *< .05.

## 3 RESULTS

### 3.1 USP35 knockdown inhibits lung cancer cell growth, colony formation, and tumor progression

**FIGURE 1:**
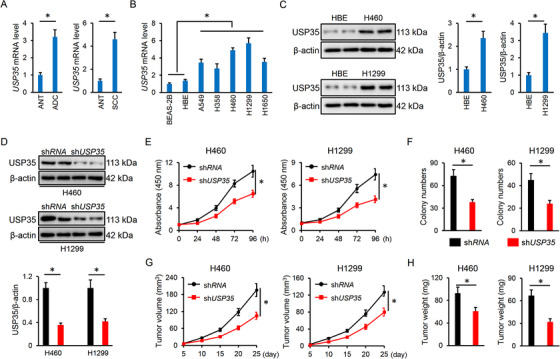
USP35 knockdown inhibits lung cancer cell growth, colony formation and tumor progression. A, Relative *USP35* mRNA level in ADC, SCC and corresponding ANT tissues (n = 10). B, Relative *USP35* mRNA level in normal human lung epithelial cell lines and lung cancer cell lines (n = 6). C, Representative immunoblots of USP35 and the quantitative data (n = 6). D, Relative USP35 protein level in lung cancer cell lines with or without sh*USP35* infection (n = 6). E, Cell viability data from CCK‐8 assay (n = 5). F, Colony formation (n = 6). G, Tumor volumes at indicating times in tumor xenografts models (n = 6). H, Tumor weights at day 25 after cell inoculation (n = 6). Data are shown as mean ± SD, *P < .05 versus the matched group

We first detected the alteration of USP35 abundance in lung cancer tissues and cell lines. As shown in Figure 1A, USP35 was significantly upregulated in both lung ADC and SCC tumors, as confirmed by the increased *USP35* mRNA levels. Accordingly, *USP35* mRNA levels were also highly expressed in lung cancer cell lines (A549, H358, H460, H1299, and H1650) compared to the normal BEAS‐2B and HBE lung epithelial cell lines (Figure 1B). Given the higher *USP35* mRNA abundance in H460 and H1299 cells, we used these two cell lines in the next study. In line with the mRNA levels, USP35 proteins were also increased in H460 and H1299 cells (Figure 1C). To clarify the role of USP35 in the pathogenesis of lung cancer, we silenced USP35 in H460 and H1299 cells, and the efficiency was determined by IB data (Figure 1D). Interestingly, USP35 knockdown inhibits the growth ability and colony formation of H460 and H1299 cells in vitro (Figure 1E,F and Figure S1A). Data from the tumor‐xenografts experiment further indicated that USP35 silence suppressed tumor volumes and weights after 25‐day growth (Figure 1G,H). Migration and invasion are the prerequisite for cancer cells metastasis to distant sites. Therefore, we evaluated the role of USP35 on the metastatic potential. As shown in Figure S1B,C, USP35‐deficient cells had decreased migrative and invasive capacities compared with the control group. All the findings identify a critical role of USP35 in regulating lung cancer cell growth and tumor progression.

### 3.2 USP35 knockdown promotes ferroptosis in lung cancer cells

**FIGURE 2:**
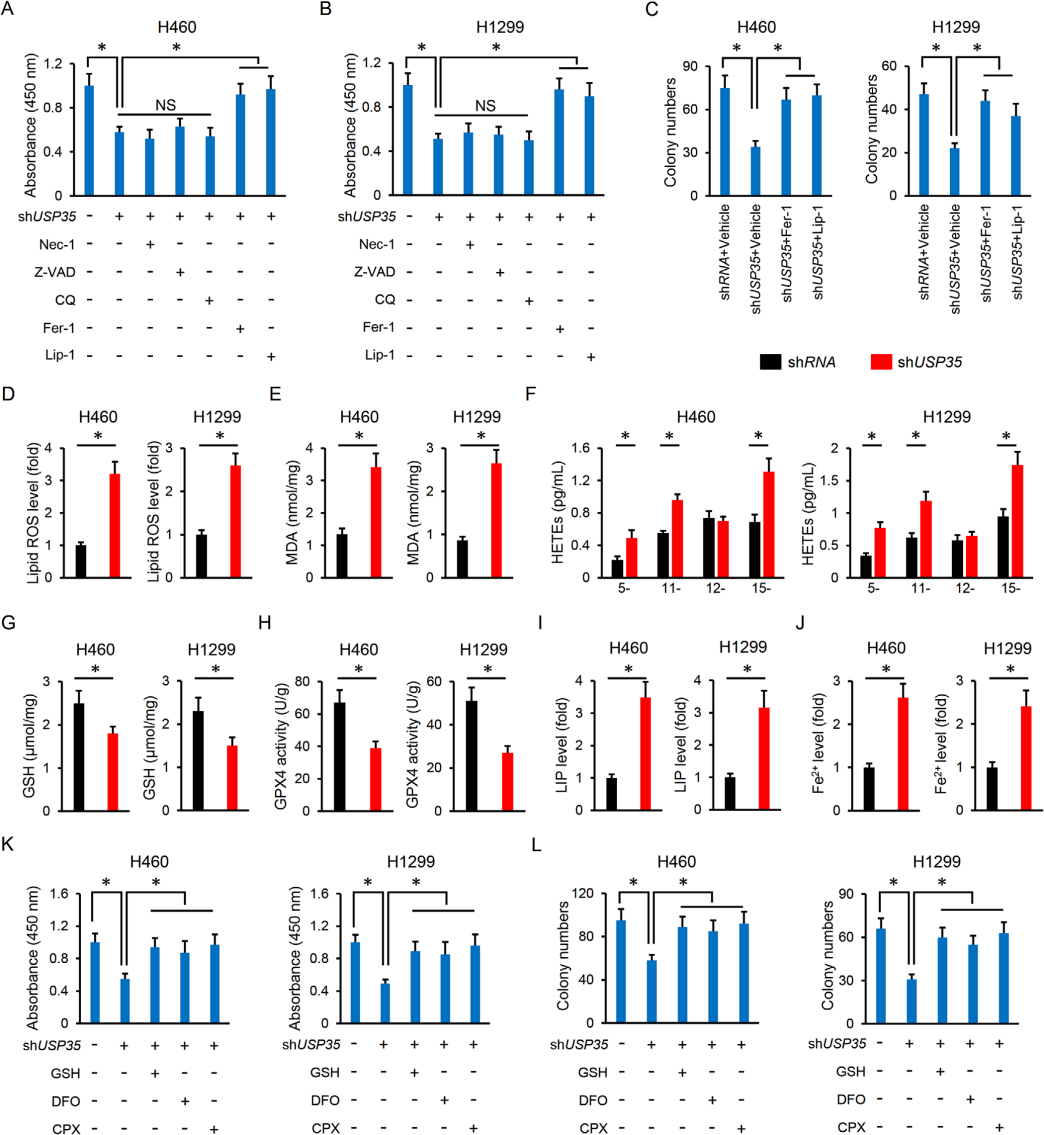
USP35 knockdown promotes ferroptosis in lung cancer cells. A,B, Cell viability data from CCK‐8 assay (n = 5). C, Colony formation (n = 6). D,E, Lipid ROS generation and MDA levels in lung cancer cells with or without USP35 silence (n = 6). F, Relative levels of HETEs released to the medium (n = 6). G,H, GSH levels and GPX4 activities in H460 and H1299 cell lines with or without USP35 silence (n = 6). I,J, Relative LIP and Fe^2+^ levels (n = 6). K,L, Cell viability and colony formation (n = 6). Data are shown as mean ± SD, *P < .05 versus the matched group. NS indicates no significance

We next explored whether USP35 regulated cell growth and tumor progression by affecting the lung cancer cell cycle. As shown in Figure S2A, USP35 knockdown did not affect the cell numbers in G0/G1, S or G2/M phases. Accordingly, the expressions of cell cycle‐associated proteins, including p21, p27, and cyclin D1 were also unaltered (Figure S2B,C). Besides, the activation of PI3K‐AKT and MEK1/2‐ERK1/2, two important cancer signaling pathways, was also unchanged by USP35 silence in lung cancer cells (Figure S2D,E). We then investigated whether sh*USP35*‐mediated tumor inhibition could be attributed to the induction of cell death. As shown in Figure 2A,B, the treatment with Nec‐1, Z‐VAD and CQ to inhibit necroptosis, apoptosis and autophagy did not affect cell viability in sh*USP35*‐infected cells, indicating that other forms of cell death may be involved. Mitophagic cell death was implicated in the progression of lung cancer and Wang et al previously determined that USP35 overexpression could inhibit mitophagy.^61^, ^62^ We then used two different mitophagy inhibitors to evaluate whether sh*USP35* infection decreased cell viability through the induction of mitophagy. As shown in Figure S2F, neither Mdivi‐1 nor CsA treatment affected cell viability upon sh*USP35* infection. Ferroptosis is a novel regulated cell death and differs from classical necrosis, apoptosis, autophagy or mitophagy, which is implicated in the pathogenesis of tumor growth, including lung cancer.^7^, ^9^ We thus explored whether ferroptosis contributed to lung cell death after USP35 silence using two kinds of ferroptosis inhibitors, Fer‐1 and Lip‐1. As depicted in Figure 2A,B, ferroptosis inhibition blocked sh*USP35*‐mediated cell death in H460 and H1299 cells. Colony formation was inhibited in sh*USP35*‐infected cells, but abolished by Fer‐1 and Lip‐1 (Figure 2C; Figure S3A). Iron‐dependent accumulation of toxic lipid‐based ROS is the critical pathogenic factor during ferroptosis.^9^, ^14^ Consistently, USP35 knockdown increased lipid ROS generation and MDA production (Figure 2D). Excessive ROS caused PUFAs oxidative damage and fragmentation into various products, including HETEs. We observed that USP35 silence promoted the releases of 5‐HETE, 11‐HETE, and 15‐HETE from H460 and H1299 cells without affecting 12‐HETE releases (Figure 2F). GSH/GPX4‐based ROS scavenging mechanism plays indispensable roles in preventing lipid peroxidation during ferroptosis.^12^ Unfortunately, GSH levels were decreased in *USP35*‐deficient cells, accompanied by the inhibition on GPX4 activities (Figure 2G,H). The intracellular LIP and Fe^2+^ are a pool of redox‐active iron that directly facilitate the formation of free radicals to trigger oxidative damage.^14^, ^32^ As shown in Figure 2I,J, labile iron and Fe^2+^ levels were significant higher in sh*USP35*‐infected cells than the controls. In contrast, GSH supplementation or iron chelators notably attenuated cell death and colony formation in sh*USP35*‐infected cells (Figure 2K,L; Figure S3B). Therefore, we conclude that ferroptosis contributes to USP35 knockdown‐induced cell death in lung cancer cells.

### 3.3 USP35 overexpression blocks erastin/RSL3‐mediated tumor suppressive effects

**FIGURE 3:**
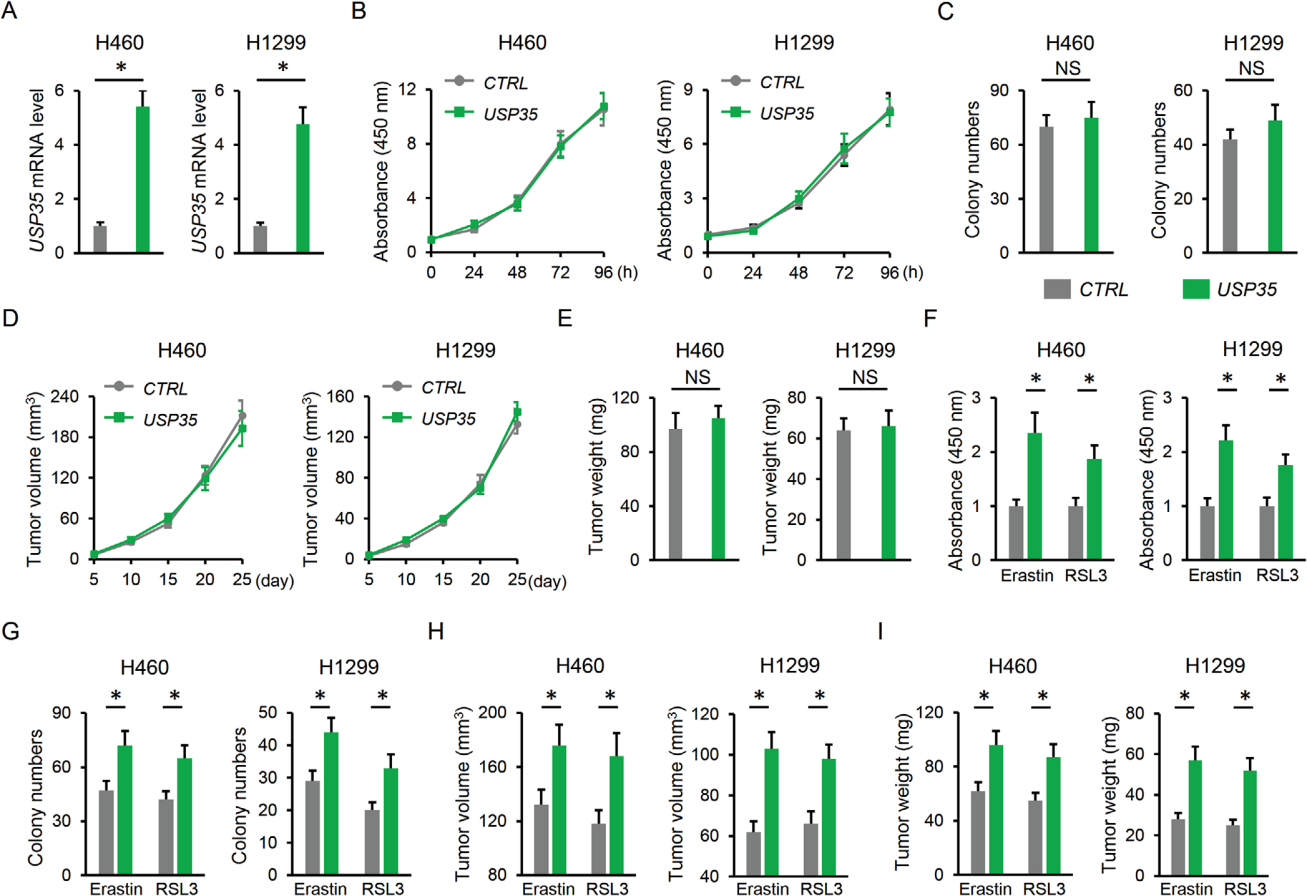
USP35 overexpression blocks erastin/RSL3‐mediated tumor suppressive effects. A, Relative *USP35* mRNA level in lung cancer cell lines with or without USP35 overexpression (n = 6). B,C, Cell viability and colony formation (n = 6). D, Tumor volumes at indicating times in tumor xenografts models (n = 6). E, Tumor weights at day 25 after cell inoculation (n = 6). F,G, Cells were incubated with *USP35* at a MOI of 10 or the *CTRL* for 12 h, and then stimulated with erastin (5 µmol/L) or RSL3 (2 µmol/L) for additional 96 h after the removal of lentiviral vectors. Cell viability and colony formation in lung cancer cell lines were determined (n = 6). H,I, Cells with or without USP35 overexpression were subcutaneously injected into the right dorsal flank of the nude mice. To induce ferroptosis in vivo, the tumor‐bearing mice were treated with erastin (15 mg/kg twice every other day) by intraperitoneal injections or RSL3 (100 mg/kg twice a week) via intratumoral injections from day 18 after cell inoculation. Tumor volumes and weights were determined at day 25 after cell inoculation (n = 6). Data are shown as mean ± SD, *P < .05 versus the matched group. NS indicates no significance

The cells were also infected with lentiviral vectors to overexpress USP35 in vitro and the efficiency was verified by PCR data (Figure 3A). Yet, USP35 overexpression did not affect cell death and colony formation in H460 and H1299 cells under basal conditions (Figure 3B,C; Figure S3C). The tumor volumes and weights in nude mice were also unaffected by USP35 overexpression (Figure 3D,E; Figure S3D). We further investigated whether USP35 manipulation had an impact on cell death upon ferroptotic stimulation. As expected, erastin or RSL3 treatment significantly reduced cell viability or colony formation in H460 and H1299 cells, which were prevented by USP35 overexpression (Figure 3F,G). Correspondingly, the nude mice inoculated with *CTRL* lung cancer cells had decreased tumor volumes and weights after erastin or RSL3 treatment; however, these tumor suppressive effects were blocked with *USP35* overexpression (Figure 3H,I). Collectively, the data indicate that USP35 overexpression blocks erastin/RSL3‐mediated tumor suppressive effects.

### 3.4 USP35 overexpression reduces erastin/RSL3‐triggered iron disturbance and ferroptosis

**FIGURE 4:**
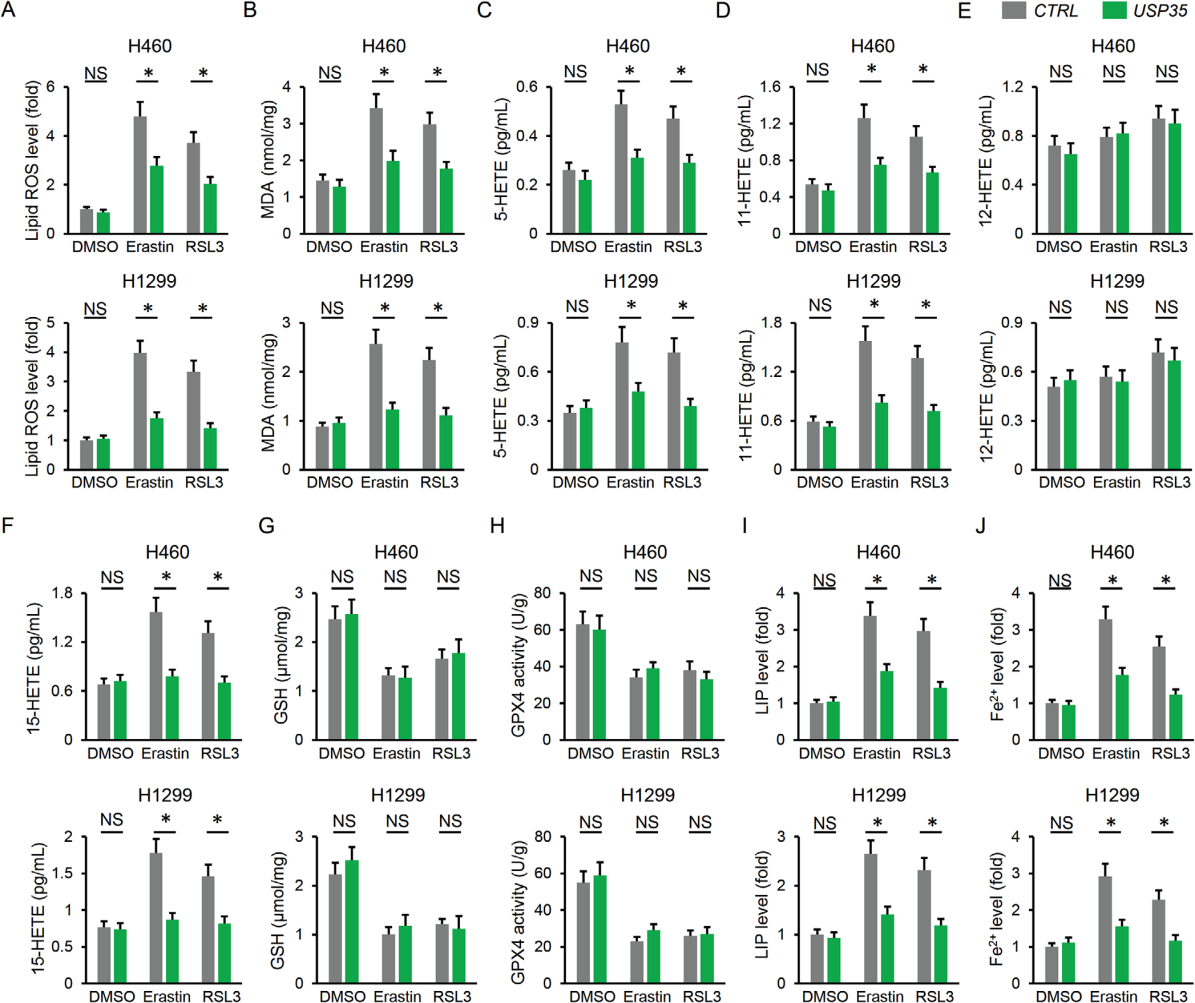
USP35 overexpression reduces erastin/RSL3‐triggered iron disturbance and ferroptosis. A,B, Lipid ROS generation and MDA levels in lung cancer cells (n = 6). C,F, Statistical data about the releases of arachidonic acid metabolites (5‐HETE, 11‐HETE, 12‐HETE, and 15‐HETE) in the cell culture medium using LC–MS/MS analysis (n = 6). G,H, GSH levels and GPX4 activities in H460 and H1299 cell lines (n = 6). I,J, Relative LIP and Fe^2+^ levels (n = 6). Data are shown as mean ± SD, *P < .05 versus the matched group. NS indicates no significance

**FIGURE 5:**
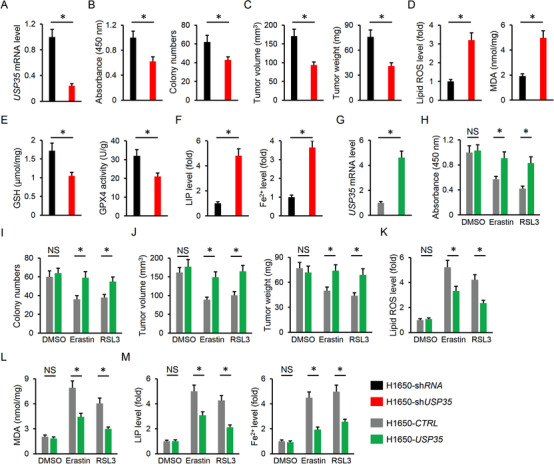
USP35 modulates cell growth, colony formation, tumor progression, and ferroptosis in EGFR mutated H1650 cells. A, Relative *USP35* mRNA level in H1650 cells with or without USP35 silence (n = 6). B, Cell viability and colony formation (n = 6). C, Tumor volumes and tumor weights at day 25 after cell inoculation (n = 6). D, Lipid ROS generation and MDA levels in H1650 cells with or without USP35 silence (n = 6). E, GSH levels and GPX4 activities in H1650 cells with or without USP35 silence (n = 6). F, Relative LIP and Fe^2+^ levels (n = 6). G, Relative *USP35* mRNA level in H1650 cells with or without USP35 overexpression (n = 6). H,I, Cell viability and colony formation (n = 6). J, Tumor volumes and tumor weights at day 25 after cell inoculation (n = 6). K,L, Lipid ROS generation and MDA levels in H1650 cells (n = 6). M, Relative LIP and Fe^2+^ levels (n = 6). Data are shown as mean ± SD, *P < .05 versus the matched group. NS indicates no significance

As expected, erastin and RLS3‐elicited lipid ROS and MDA generation were decreased by USP35 overexpression (Figure 4A,B). Besides, HETEs levels were increased in erastin and RLS3‐treated cancer cells, but were decreased in those with USP35 overexpression except 12‐HETE (Figure 4C,F). However, USP35 overexpression made no alteration on GSH contents and GPX4 activities (Figure 4G,H). As depicted in Figure 4I,J, USP35 overexpression significantly blunted the induction of LIP and Fe^2+^ in H460 and H1299 cells with erastin or RSL3 stimulation. In line with the phenotypic alteration, USP35 overexpression also had no impact on iron metabolism and ferroptosis under basal conditions (Figure 4A,J). As mentioned above, both H460 and H1299 cells have high basal USP35 expression, we then checked the effect of USP35 overexpression in normal cells and USP35 low expressing lung cancer cells. USP35 overexpression in BEAS‐2B and HBE normal human lung epithelial cell lines was clarified by PCR assay (Figure S4A). As depicted in Figure S4B,C, USP35 overexpression did not affect cell viability or migration of the BEAS‐2B and HBE cells. In addition, cell growth, colony formation, migration, and invasion were also unaltered by USP35 overexpression in USP35 low expressing A549 and H358 cells (Figure S4D‐H). H460 and H1299 cells are epidermal growth factor receptor (EGFR) wild‐type cells and we further determined the role of USP35 on cell growth, ferroptosis induction, and tumor growth in EGFR mutated H1650 cells. As show in Figure 5A‐C, USP35 silence significantly reduced H1650 cell growth, colony formation and tumor progression. Accordingly, lipid ROS generation, MDA production, intracellular LIP and Fe^2+^ were increased, while GSH levels and GPX4 activities were decreased in *USP35*‐deficient H1650 cells (Figure 5D‐F). Conversely, USP35 overexpression notably prevented erastin/RSL3‐induced suppression on H1650 cell growth, colony formation and tumor progression in vitro and in vivo (Figure 5G‐J). The increased lipid ROS generation, MDA production and iron load were also reduced by USP35 overexpression in H1650 cells (Figure 5K‐M). Overall, our findings suggest that USP35 overexpression reduces erastin/RSL3‐triggered iron disturbance and ferroptosis.

### 3.5 USP35 modulates ferroptosis via targeting FPN

**FIGURE 6:**
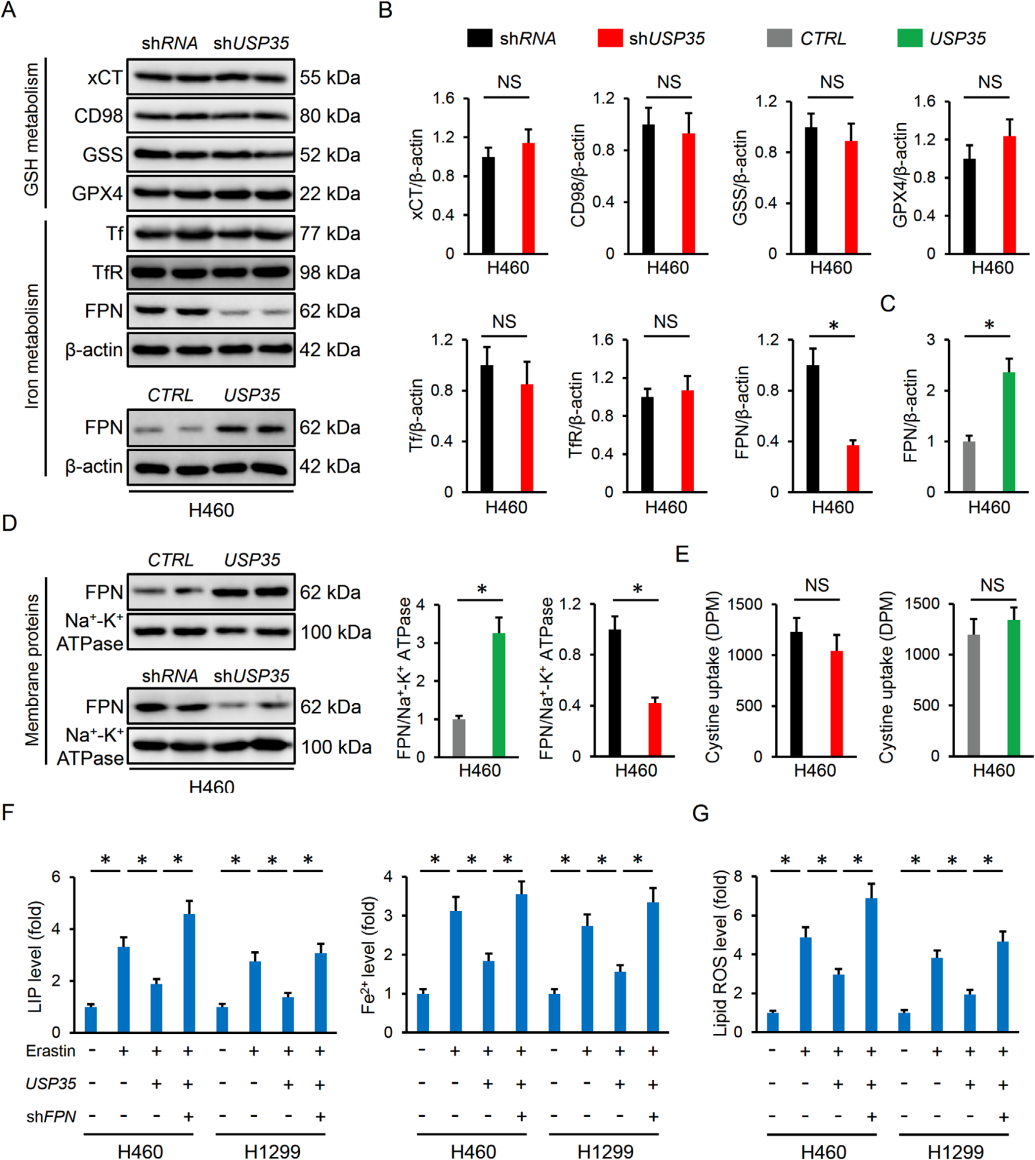
USP35 modulates ferroptosis via targeting FPN. A‐D, Representative immunoblots and the quantitative data (n = 6). E, Cystine uptake levels (n = 6). F, Relative LIP and Fe^2+^ levels (n = 6). G, Lipid ROS generation (n = 6). Data are shown as mean ± SD, *P < .05 versus the matched group. NS indicates no significance

**FIGURE 7:**
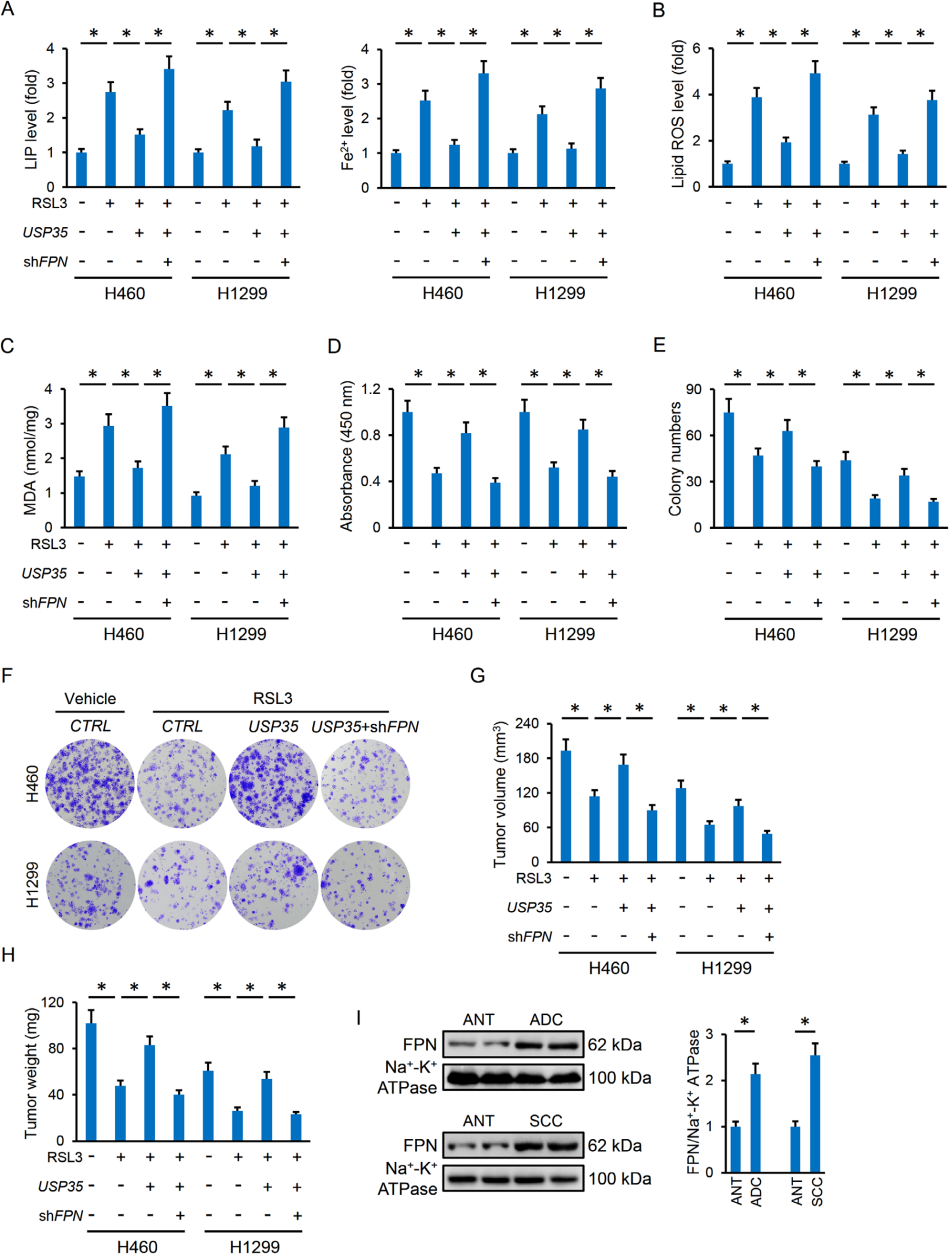
USP35 overexpression inhibits RSL3‐induced ferroptosis and tumor progression via targeting FPN. A, Relative LIP and Fe^2+^ levels (n = 6). B,C, Lipid ROS generation and MDA levels in lung cancer cells (n = 6). D‐F, Cell viability and colony formation (n = 6). G‐H, Tumor volumes and tumor weights in tumor xenografts models (n = 6). I, Representative immunoblots and the quantitative data (n = 6). Data are shown as mean ± SD, *P < .05 versus the matched group

We next tried to elucidate the possible mechanisms of USP35 on ferroptosis. As shown in Figure 5A,B, USP35 silence did not affect the protein expressions involved in GSH uptake (xCT and CD98) and synthesis (GSS). These data might partially explain the unaltered GSH and GPX4 activities in *USP35*‐overexpressed cells. As for the decreased levels of GSH contents and GPX4 activities in sh*USP35*‐infected cells, they could be attributed to ferroptosis‐dependent depletion. Besides, the proteins responsible for iron import (Tf and TfR) were also unaltered; however, the pivotal iron transport protein in mammals, FPN was decreased in *USP35*‐deficient cells and increased in *USP35*‐overexpressed cells (Figure 6A‐C). Particularly, FPN protein abundances in cell membrane were increased in cancer cells with USP35 overexpression, but decreased by USP35 knockdown (Figure 6D). In line with the molecular alteration, cystine uptake was also unaffected in cells with USP35 silence or overexpression (Figure 6E). To further confirm the involvement of FPN, H460 and H1299 cells were preinfected with sh*FPN* respectively to knock down endogenous FPN expression, and the efficiency was provided in Figure S5A. As mentioned above, USP35 overexpression decreased intracellular LIP and Fe^2+^ in lung cancer cells upon ferroptotic stimulation, yet failed to do so in *FPN*‐deficient cells (Figure 6F). Besides, the decreased generations of lipid ROS and MDA by USP35 overexpression were also retarded after knocking down endogenous FPN (Figure 6G; Figure S5B). Consistently, USP35 lost its motivated effects on cell viability and colony formation upon erastin stimulation in lung cancer cells with sh*FPN* infection (Figure S5C,D). The enhanced tumor growth‐derived from *USP35*‐overexpressed cells was also inhibited by FPN silence (Figure S5E,F). We also assessed the role of FPN in lung cancer cells or tumor xenografts upon RSL3 treatment in vivo and in vitro. As shown in Figure 7A‐C, FPN knockdown restored intracellular LIP and Fe^2+^ levels in USP35‐overexpressed lung cancer cells, accompanied by increased formations of lipid ROS and MDA. Meanwhile, increased cell viability and colony formation by USP35 overexpression were also reversed after FPN knockdown (Figure 7D‐F). In line with the in vitro data, USP35‐overexpressed cells had decreased tumor volumes and weights after FPN knockdown (Figure 7G,H). More importantly, we found that the membrane distribution of FPN was notably increased in lung ADC and SCC tumors (Figure 7I). Taken together, these data identified FPN as a potential target of USP35 in modulating ferroptosis and tumor progression.

### 3.6 USP35 is required for FPN protein stability in lung cancer cells

**FIGURE 8:**
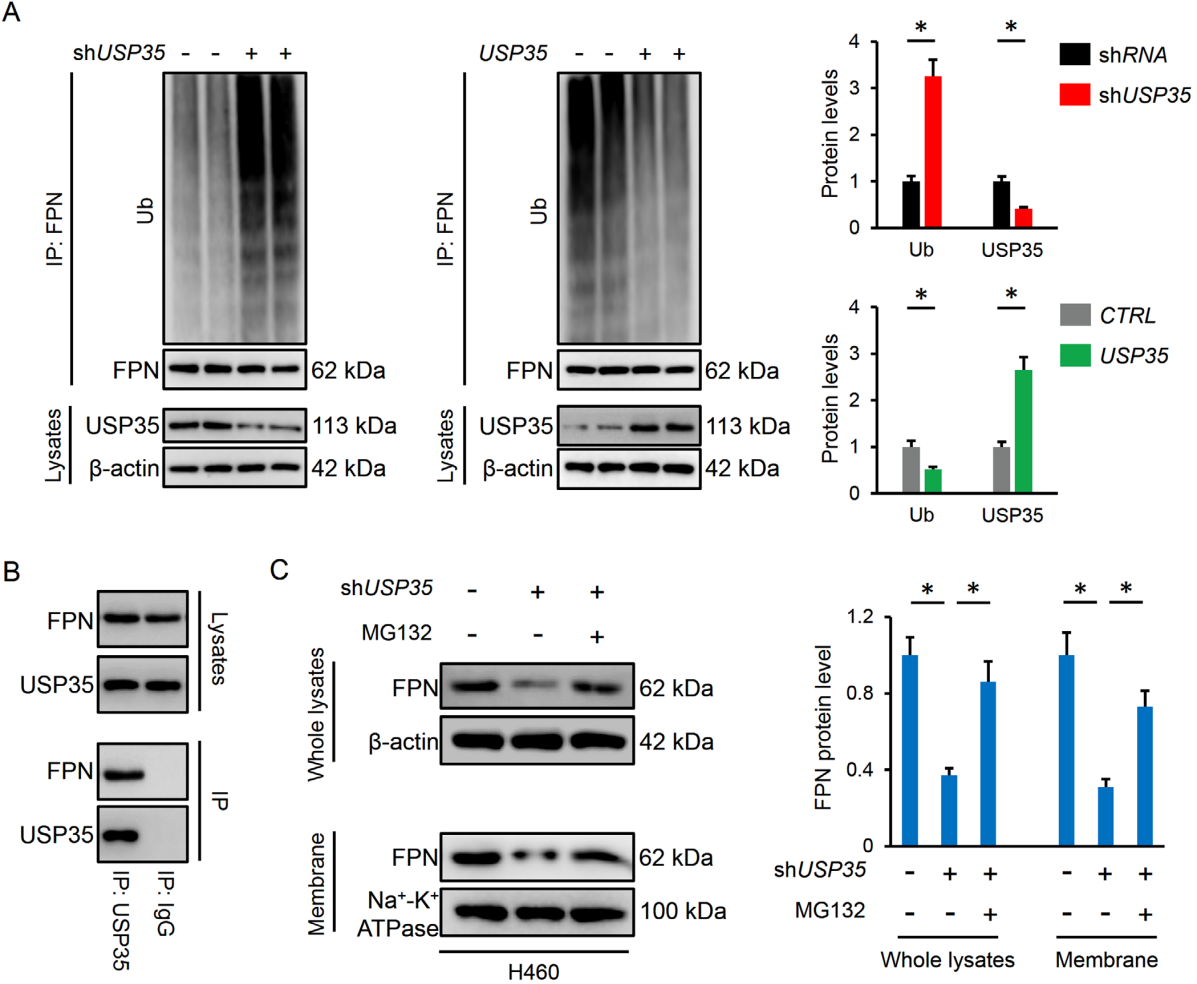
USP35 is required for FPN protein stability in lung cancer cells. A, Relative ubiquinated levels of FPN in lung cancer cells with or without USP35 manipulation (n = 6). B, FPN expression in the whole lysates or cell membrane from sh*USP35*‐infected cells with or without MG132 treatment (n = 6). C, Endogenous interaction between USP35 and FPN via the IP assay (n = 4). Data are shown as mean ± SD, *P < .05 versus the matched group

USP35 belongs to the DUBs family and plays critical roles in controlling Ub‐dependent degradation of various proteins.^24^, ^26^ In addition, FPN ubiquitination and subsequent endocytosis contribute to iron overload and ferroptosis.^63^ Therefore, we investigated whether USP35 regulated FPN expression via affecting its protein stability. As shown in Figure 8A, USP35 knockdown increased, while USP35 overexpression decreased the ubiquitinated FPN levels. And the decreased FPN levels in whole or membrane lysates from sh*USP35*‐infected cells were prevented upon treatment with the MG132 proteasome inhibitor (Figure 8B). Subsequently, we examined whether USP35 is a direct binding partner of FPN. The data by IP assay revealed the association between endogenous USP35 and FPN (Figure 8C). Together, these data indicate that USP35 can directly interact with FPN and functions as a deubiquitinase to maintain its protein stability.

### 3.7 USP35 knockdown enhanced the chemotherapeutic sensitivity of lung cancer cells

**FIGURE 9:**
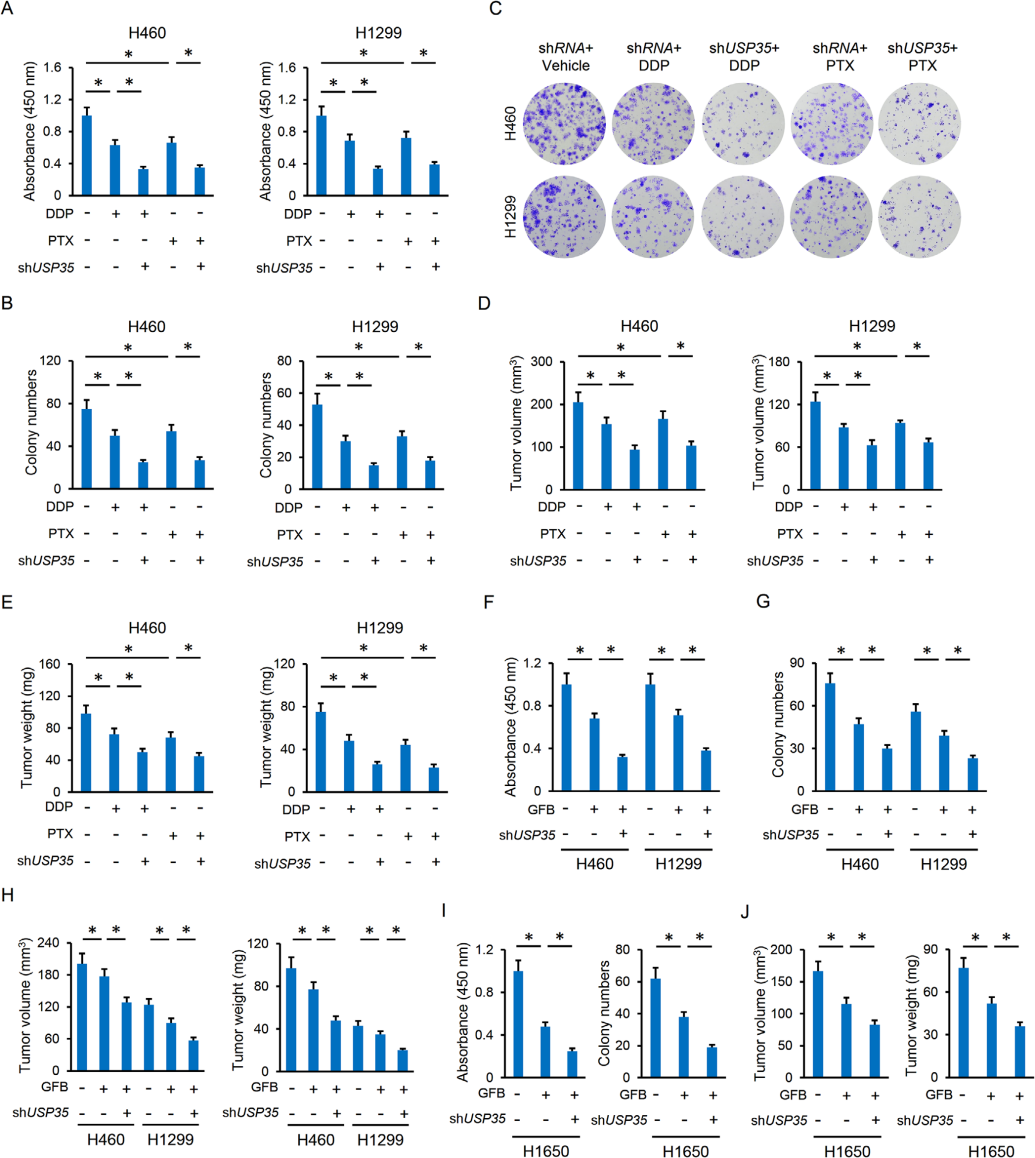
USP35 knockdown enhanced the chemotherapeutic sensitivity of lung cancer cells. A‐C, Cell viability and colony formation (n = 6). D‐E, Tumor volumes and tumor weights in tumor xenografts models (n = 6). F‐G, Cell viability and colony formation of the H460 or H1299 cell lines (n = 6). H, Tumor volumes and tumor weights in tumor xenografts models of the H460 or H1299 cell lines (n = 6). I, Cell viability and colony formation of the H1650 cells (n = 6). J, Tumor volumes and tumor weights in tumor xenografts models of the H1650 cells (n = 6). Data are shown as mean ± SD, *P < .05 versus the matched group

Given the high expression of USP35 in lung cancer cells and its role in regulating ferroptosis, we finally evaluated whether USP35 silence could sensitize lung cancer cells to the chemotherapeutic drugs. As depicted in Figure 9A‐C, *USP35*‐deficient H460 and H1299 cells were more sensitive to the toxic effects of DDP and PTX in vitro, as evidenced by the decreased cell viability and colonization. Correspondingly, the tumor‐bearing mice inoculated with *USP35*‐deficient cancer cells had reduced tumor volumes and weights upon DDP or PTX treatment (Figure 9D,E). Tyrosine kinase inhibitors (TKI) have emerged as an alternative to conventional chemotherapy and provide dramatic survival benefits to lung cancer patients. We then measured the synergistic effects between USP35 knockdown and TKI in H460 and H1299 cells, and the data suggested that USP35 silence sensitized H460 and H1299 cells to GFB chemotherapy in vivo and in vitro (Figure 9F‐H). Of note, EGFR‐mutated lung cancer cells (eg, H1650 cell) are more sensitive to TKI, and we thus evaluated whether USP35 silence would sensitize H1650 cell to GFB chemotherapy. As shown in Figure 9I,J, USP35 silence further suppressed the growth, colony formation and tumor progression of H1650 cells upon GFB treatment. Overall, our data prove that *USP35*‐deficient lung cancer cells are more sensitive to chemotherapeutic drugs.

## 4 DISCUSSION

The present study identify the role and potential molecular basis of USP35 on ferroptosis in lung cancer cells. USP35 is abundant in human lung cancer tissues and cell lines compared with the ANT or normal lung epithelial cells. Knockdown of USP35 facilitates FPN ubiquitination and degradation, and decreases FPN‐dependent iron export, thereby triggering iron overload and ferroptosis in lung cancer cells. Besides, we observe that USP35 overexpression does not affect ferroptosis and tumor progression under basal conditions, but results in an alleviation on erastin/RSL3‐caused iron disturbance and ferroptosis, accompanied by the increased lung cancer cell growth and tumor progression. Moreover, USP35 knockdown sensitizes lung cancer cells to DDP and PTX chemotherapy, defining USP35 as a promising therapeutic target to lung cancer.

Ferroptosis functions as a novel non‐apoptotic programmed cell death and is proposed as a promising therapeutic target to lung cancer. Chen et al proved that stimulating ferroptosis inhibited the growth and migration of lung cancer cells, whereas Wang et al demonstrated that ferroptosis suppression facilitated cell growth, colonization and tumor formation in lung cancer cells.^7^, ^30^ In addition, targeting ferroptosis provides novel insights to understand the therapeutic mechanisms of antitumor drugs that is essential for developing new‐generation chemotherapeutic agents for lung cancer.^30^, ^64^ Interestingly, ferroptotic agonist can decrease radioresistance of non‐small cell lung cancer cells.^65^ Consistently, we herein found that USP35 silence promoted ferroptosis of lung cancer cells, thereby sensitizing them to DDP and PTX chemotherapy in vivo and in vitro. Iron is a necessary trace element for normal cellular function and organic health, and dysregulation of intracellular iron homeostasis closely correlates with the development of several malignant tumors, including lung cancer.^14^ As we know, iron is an important executor of ferroptosis, and LIP, primarily referred as the Fe^2+^, is especially vital for the provocation of ferroptotic cell death via triggering cytotoxic lipid radicals overproduction.^9^, ^32^ Intracellular iron levels are orchestrated by iron regulating transporters. FPN is the only known mammalian iron exporting protein and is required for systemic iron homeostasis via mediating duodenal iron releases into the circulation.^15^ However, emerging studies suggest that FPN is also expressed in various cancer cells and plays critical roles in maintaining cellular iron homeostasis, including the lung cancer cells. Results from Babu et al showed that FPN was decreased in human lung ADC and SCC tumors, accompanied by the decreased iron export and increased intracellular iron retention.^66^ Correspondingly, our findings revealed that FPN suppression increased intracellular iron levels and ferroptosis, thereby decreasing lung cancer cell growth and tumor progression.

USP35 belongs to the DUBs family that is associated with cell proliferation and mitosis.^24^, ^25^ Zhang et al recently found that USP35 was upregulated in ovarian cancer tissues and that the high USP35 expression correlated with a poor prognosis in ovarian cancer patients. While *USP35*‐deficient ovarian cancer cells were sensitized to DDP chemotherapy.^26^ In the current study, we also detected a high USP35 expression in human lung cancer tissues and cell lines. Such USP35 abundance did not affect lung cancer cell growth, colonization, and tumor formation under basal conditions, but prevented erastin/RSL3‐elicited ferroptosis and tumor‐suppressive effects. In contrast, knockdown of endogenous USP35 expression increased intracellular iron levels and provoked ferroptosis to inhibit lung cancer cell growth, colony formation and tumor progression. We further evaluated the effect of USP35 silence on the chemosensitivity of lung cancer cells to DDP and PTX, and found that *USP35*‐deficient lung cancer cells are more sensitive to DDP and PTX chemotherapy. FPN ubiquitination is required for its internalization and degradation, and Zhang et al identified the deubiquitinated role of USP35 in ovarian cancer cells.^26^, ^63^ Consistently, we found that USP35 directly interacted with FPN and functioned as a deubiquitinase to maintain its protein stability. Of course, there are some limitations in the present study. First, the specific domain through which USP35 interacts with FPN remains unclear and demands further investigation. Zhang et al recently reported that the C‐terminal USP domains, rather than the N‐terminal HEAT repeats region of USP35 were required for its interaction with STING. However, whether this domain mediating the interaction with FPN needs further verification. Besides, representative transcriptome and/or proteome experiments will help to identify the upstream and downstream ferroptotic signalling pathways.

Collectively, our data prove that USP35 is required for FPN protein stability and the iron export in lung cancer cells, thereby preserving intracellular iron homeostasis and tumor growth. While USP35 knockdown increases intracellular LIP levels and ferroptosis via decreasing FPN‐mediated iron export, accompanied by the decreases of lung cancer cell growth, colonization and tumor formation, and the increases of chemosensitivity to DDP and PTX.

## ETHICS APPROVAL AND CONSENT TO PARTICIPATE

All animal experiments were performed in accordance with the *Animal Research: Reporting of In Vivo Experiments* (ARRIVE) guidelines and also approved by the Animal Ethics Committee of Zhongnan Hospital of Wuhan University. Written informed consent was obtained from the patients and families of the donors.

## CONFLICT OF INTEREST

The authors declare no conflicts of interest.

## Supporting information

Supporting Information

## Data Availability

All data that support the findings in this study are available from the corresponding author upon reasonable request.
