# Supplementary material for: Deubiquitinase USP35 modulates ferroptosis in lung cancer via targeting ferroportin
Source: Clin Transl Med. 2021 May 1;11(4):e390. doi: 10.1002/ctm2.390 (PMC8087931; doi:10.1002/ctm2.390)
Supplement: Supplementary file 1 — Supporting Information [file CTM2-11-e390-s001.docx]

**Deubiquitinase USP35 modulates ferroptosis in lung cancer via targeting ferroportin**

Zheng Tang^1 *^, Wanli Jiang^2 *^, Ming Mao^1 #^, Jinping Zhao^1^, Jiakuan Chen^1^, Nitao Cheng^1^

^1^ Department of Thoracic Surgery, Zhongnan Hospital of Wuhan University, Wuhan 430071, China

^2^ Department of Thoracic Surgery, Renmin Hospital of Wuhan University, Wuhan, 430060, China

* These authors contributed equally to this work

^#^ Corresponding author: Prof. Ming Mao

Department of Thoracic Surgery,

Zhongnan Hospital of Wuhan University,

No.169 Donghu Road, Wuchang District, Wuhan, 430071, China

E-mail: [maoming@znhospital.com](mailto:maoming@znhospital.com)


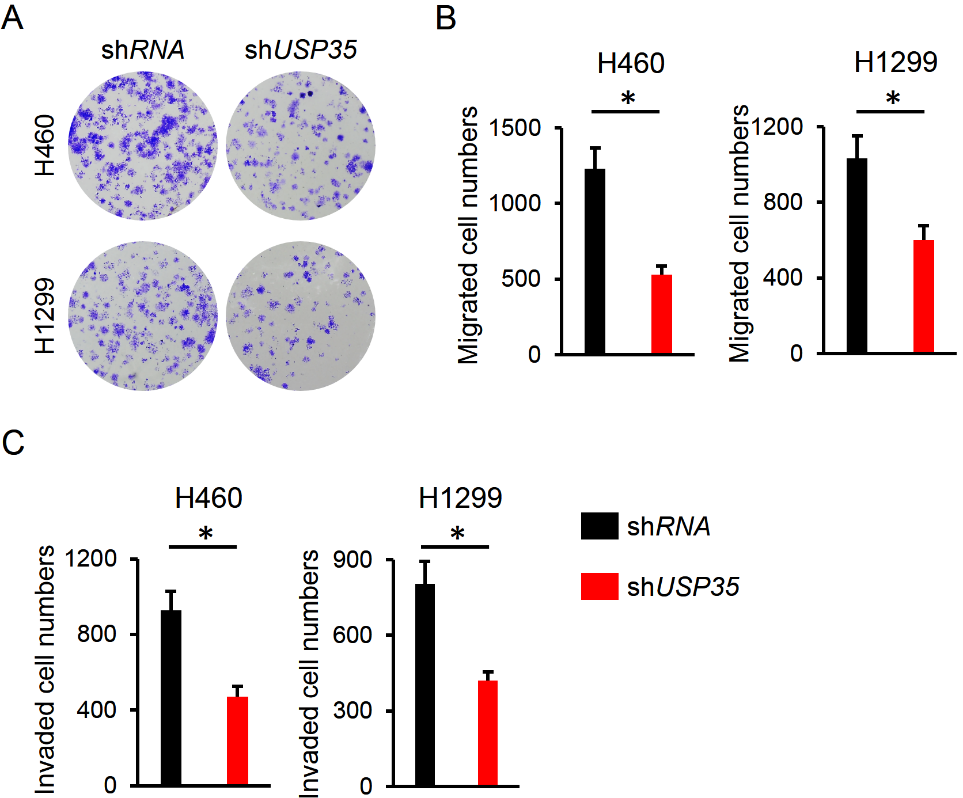


**Figure S1. USP35 knockdown inhibits lung cancer cell growth, colony formation, migration, invasion and tumor progression. (A)** Representative microscopic pictures of colony formation in Figure 1F (n=6). **(B-C)** Quantitative data about cell migration and invasion in lung cancer cell lines with or without sh*USP35* infection (n=6). Data are shown as mean ± SD, **P* < 0.05 versus the matched group.


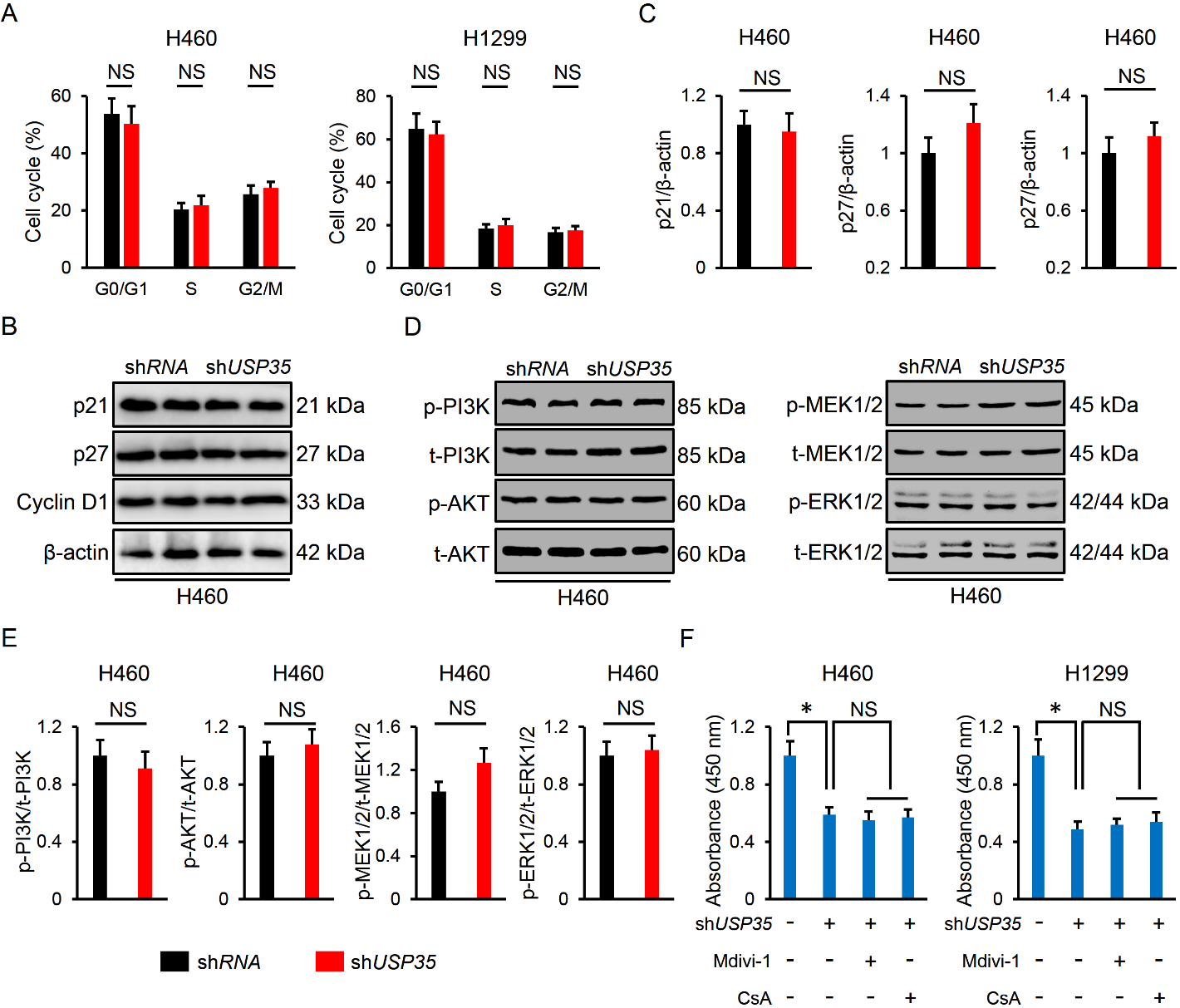


**Figure S2. USP35 silence does not affect the lung cancer cell cycle, PI3K-AKT, MEK1/2-ERK1/2 pathways and mitophagic cell death. (A)** Cell cycle analysis of H460 and H1299 cells (n=6). **(B-E)** Representative immunoblots and the quantitative data (n=6). **(F)** Cell viability data from CCK-8 assay in sh*USP35*-infected H460 and H1299 cells with or without mitophagic inhibition (n=5). Data are shown as mean ± SD, **P* < 0.05 versus the matched group. NS indicates no significance.


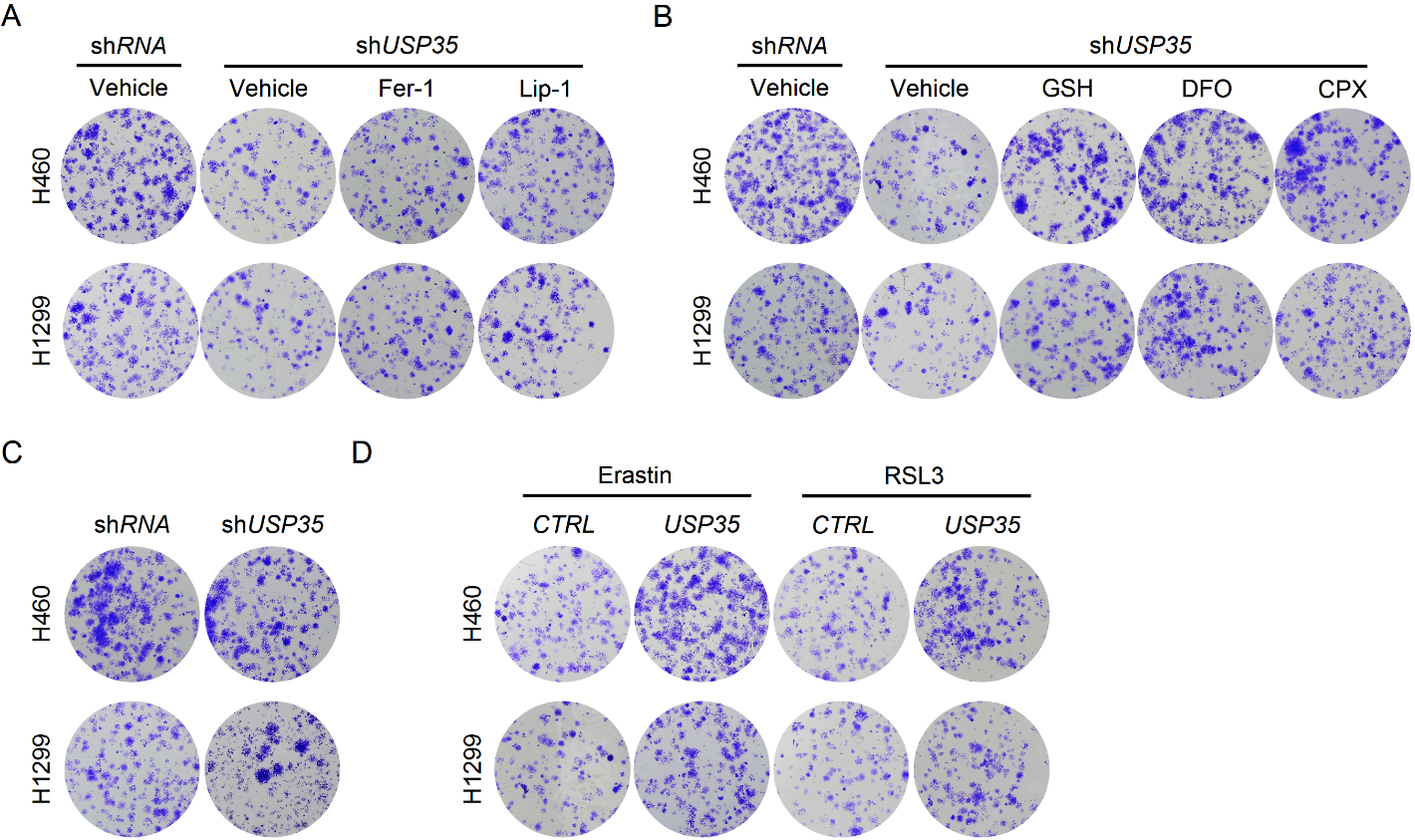


**Figure S3. USP35 knockdown promotes ferroptosis in lung cancer cells. (A-D)** Representative microscopic pictures of colony formation in Figure 2C, 2L, 3C and 3G (n=6). Data are shown as mean ± SD, **P* < 0.05 versus the matched group.


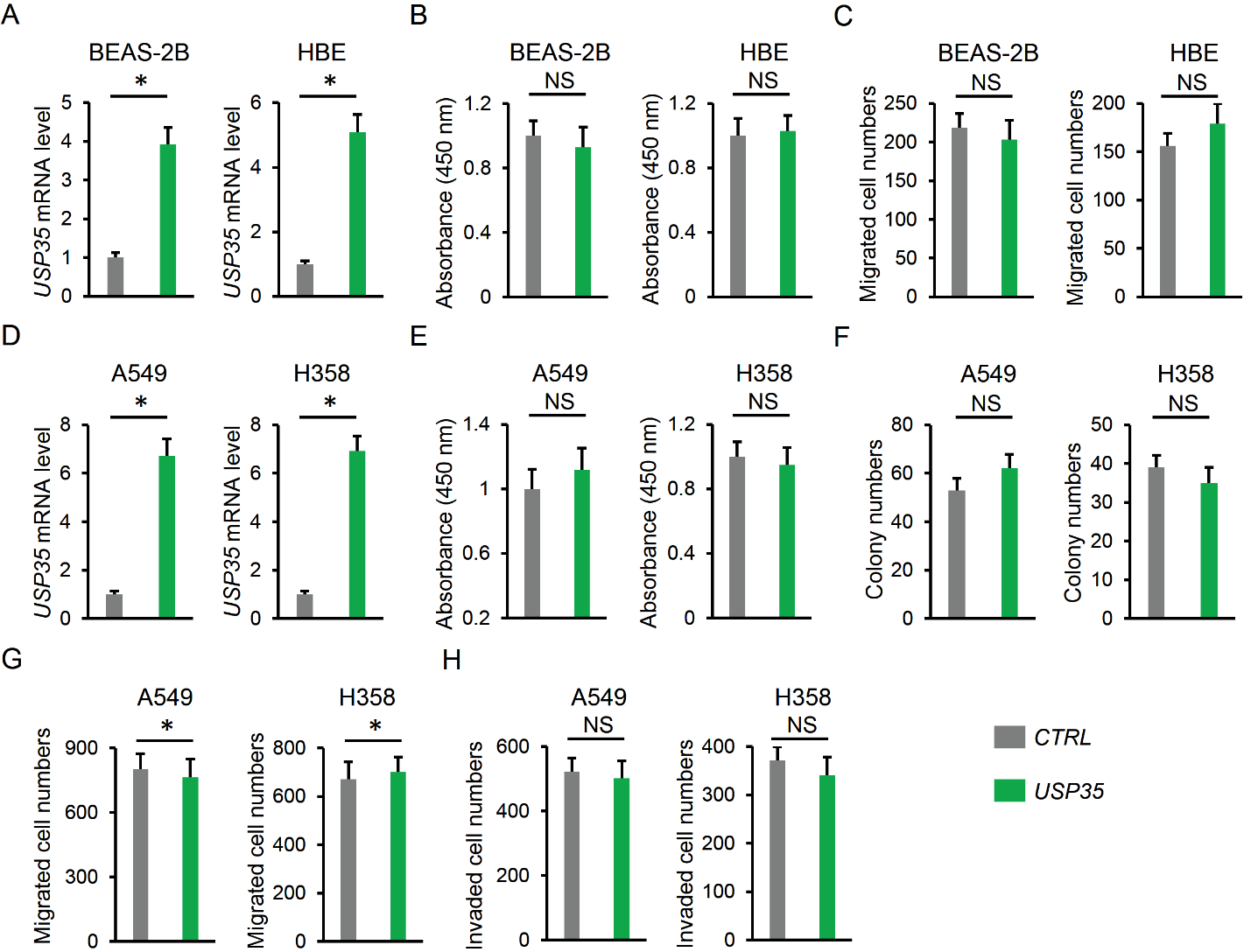


**Figure S4. USP35 overexpression does not affect cell growth, colony formation, migration and invasion in normal cells and USP35 low expressing lung cancer cells under basal conditions. (A)** Relative *USP35* mRNA level in normal human lung epithelial cell lines with or without USP35 overexpression (n=6). **(B)** Cell viability data from CCK-8 assay (n=6). **(C)** Quantitative data about cell migration in normal human lung epithelial cell lines with or without sh*USP35* infection (n=6). **(D)** Relative *USP35* mRNA level in lung cancer cell lines with or without USP35 overexpression (n=6). **(E-F)** Cell viability and colony formation (n=6). **(G-H)** Quantitative data about cell migration and invasion in lung cancer cell lines with or without sh*USP35* infection (n=6). Data are shown as mean ± SD, **P* < 0.05 versus the matched group. NS indicates no significance.


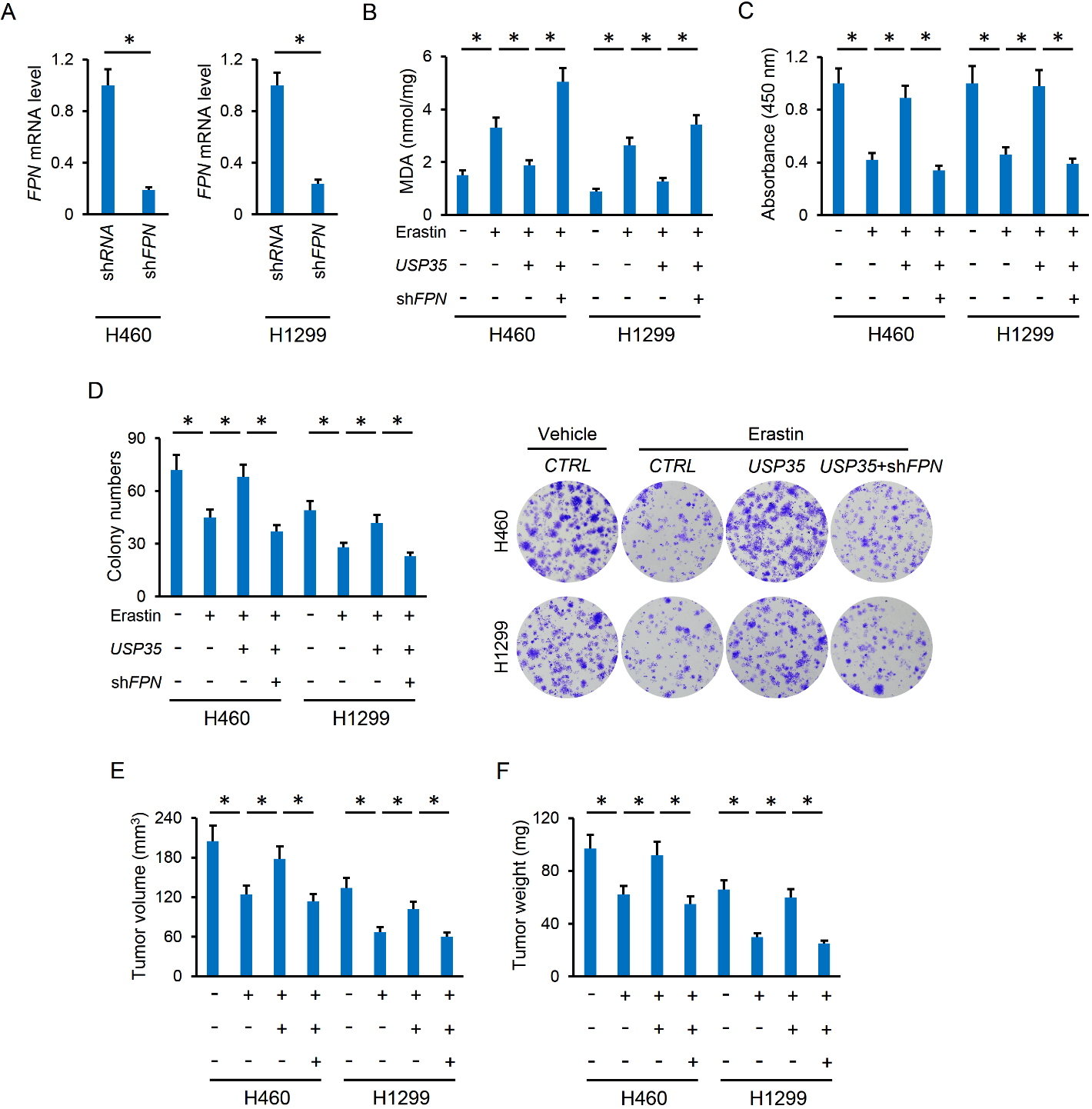


**Figure S5. USP35 overexpression inhibits erastin-induced ferroptosis and tumor progression via targeting FPN. (A)** Relative *FPN* mRNA level in lung cancer cell lines with or without sh*FPN* infection (n=6). **(B)** MDA levels in lung cancer cells (n=6). **(C-D)** Cell viability and colony formation (n=6). **(E-F)** Tumor volumes and tumor weights in tumor xenografts models (n=6). Data are shown as mean ± SD, **P* < 0.05 versus the matched group.
